# Value of dynamic contrast-enhanced magnetic resonance imaging in combination with mammography for screening early-stage breast cancer

**DOI:** 10.4314/ahs.v23i2.33

**Published:** 2023-06

**Authors:** Weiwei Su, Xiaoming Hou, Bo Yu

**Affiliations:** 1 Department of Thyroid Breast Surgery, Tongxiang First People's Hospital, Tongxiang 314500, Zhejiang Province, China; 2 Department of Radiology, Chinese PLA General Hospital, Beijing 100853, China; 3 Zhejiang Zhuji Hospital of Traditional Chinese Medicine, Zhuji 311800, Zhejiang Province, China

**Keywords:** Breast cancer, diagnosis, magnetic resonance imaging, mammography

## Abstract

**Objective:**

To study the value of dynamic contrast-enhanced magnetic resonance imaging (DCE-MRI) in combination with mammography for screening early-stage breast cancer.

**Methods:**

Ninety-three female patients visiting Zhejiang Zhuji Hospital of Traditional Chinese Medicine from January 2020 to March 2022 were enrolled to receive DCE-MRI and mammography. The diagnostic efficiencies of different methods were assessed with pathological diagnosis as the golden standard. The factors affecting diagnostic sensitivity were investigated based on clinicopathological characteristics.

**Results:**

Forty-one patients were diagnosed as malignant pathological changes by DCE-MRI, and the signs were unclear boundary with surrounding tissues and irregular or unsmooth edges. The maximum linear slope and ratio of the maximum linear Slope^R^ of malignant pathological changes were significantly larger than those of benign pathological changes (P<0.05). Forty-five patients were diagnosed as malignant pathological changes by mammography combined with DCE-MRI. Compared to single diagnosis method, the combined diagnosis had significantly increased sensitivity, specificity, accuracy, positive predictive value and negative predictive value, and decreased rates of missed diagnosis and misdiagnosis (P<0.05). Lesion diameter was an independent risk factor affecting the diagnostic sensitivity (P<0.05).

**Conclusion:**

Mammography and DCE-MRI play key roles in the early diagnosis of breast cancer, and their combination can increase the diagnostic efficiency.

## Introduction

Breast cancer, as one of the most common malignancies in females, has an increasing incidence rate in the last few years. In addition, most patients have already entered the middle- and advanced-stage upon diagnosis due to the absence of specific clinical symptoms and signs in the early stage, resulting in poor treatment outcomes and prognosis^1^,^2^. Therefore, early screening and treatment are of great significance for reducing the mortality rate of patients with breast cancer. At present, biopsy is the “gold standard” for the clinical diagnosis of breast cancer, which, however, is invasive and cumbersome in clinical application^3^.

Recently, mammography, ultrasonography (US), and dynamic contrast-enhanced magnetic resonance imaging (DCE-MRI) have been applied in the diagnosis of breast cancer in clinical practice. Mammography is highly sensitive to microcalcification in tissues and can be applied to diagnose asymptomatic breast cancer, but it has low sensitivity in diagnosing dense high-density breast and cannot show manifestations such as blood perfusion and outflow in lesion tissues^4^. Besides, the volume and density of mammary glands in Chinese women are different from those in foreign women5. Hence, the diagnostic value of mammography should be further investigated. DCE-MRI reveals more comprehensive image information through multi-plane imaging, reduces the interference of intramammary fat tissues, and has high sensitivity, but it requires a long scanning time and has low specificity in diagnosing microcalcification^6^.

DCE-MRI and mammography have their own advantages in the histomorphological examination of breast lesions. However, mammography is mainly based on the calcification in lesions, lacking the manifestations of blood flow perfusion and outflow^7^. DCE-MRI mainly relies on the enhanced performance to detect the changes of vascular tissues in lesions, which is less sensitive than mammography in disclosing the microcalcification in lesions^8^. Thus, DCE-MRI should be combined with mammography in the early diagnosis of breast cancer, which may elevate the diagnosis rate.

Thereby motivated, the value of DCE-MRI and mammography for the early screening of breast cancer, as well as related influencing factors were explored in this study, aiming to provide a reference for clinical diagnosis.

## Materials and Methods

### General data

A total of 93 patients with suspected early-stage breast cancer visiting Zhejiang Zhuji Hospital of Traditional Chinese Medicine from January 2020 to March 2022 were enrolled as subjects, with an age of 24-67 (48.54±10.36) years old. The inclusion criteria were set as follows: (1) patients with such clinical symptoms as lump, swelling pain, nipple retraction, and tangerine peel-like changes in skin in the breast, and breast mass shown on breast ultrasound images, (2) those with lesions involving unilateral breast, (3) those who had indications for mammography or DCE-MRI and agreed to perform biopsy, and (4) those who were conscious and informed of and agreed to this study. The following patients were excluded: (1) patients with other intercurrent malignant tumors, (2) hose with axillary lymph node metastasis, (3) pregnant or breastfeeding females, (4) patients with intercurrent infection or coagulation dysfunction, (5) those with insufficiency of important organs, or (6) those undergoing adjuvant therapy before surgery. This study was approved by the ethics committee of Zhejiang Zhuji Hospital of Traditional Chinese Medicine, and the enrolled subjects were informed of this study and signed the informed consent.

### Mammography method

Mammography was implemented using a digital mammography machine (Hologic Selenia, USA), with internal and external oblique and axial images routinely taken, and auxiliary tangent and local magnification were conducted if necessary. According to BI-RADS grading standard, X-ray images were enlarged for observation (mainly the observation of the morphology, location, density, edge, and calcification of mammary gland lesions). The diagnostic criteria for mammography were as follows^9^: direct signs referred to irregular fine sand-like or granular calcifications (≥5/cm^2^) arranged in clusters, high-density nodular shadows with blurred edges, burr-like manifestations and irregular borders, and focal dense shadows with uneven density. The indirect signs included local skin thickening or depression, nipple retraction or infundibular nipple, and disordered tissue structure. The presence of two direct signs or one direct sign accompanied by two indirect signs indicated breast cancer. The diagnosis was jointly made by two experienced imaging physicians.

### DCE-MRI scan

A 3.0 T superconducting magnetic resonance scanner (GE, Signa, HDx 3.0T) with a breast-specific 4-channel phased array coil was employed for DCE-MRI. A patient was in the prone position to place both breasts naturally into the coil recesses. Then conventional and dynamic enhanced scanning was carried out on both breasts. Coronal, sagittal and cross-sectional scanning was implemented routinely, and fast FSE sequence cross-sectional and sagittal T1 weighted image (T1WI) was performed according to the localization images. The scanning parameters were as follows: T1WI: TR 640 ms, TE 12 ms, slice thickness 5.0 mm, slice spacing 0.5 mm, and FOV 320 mm×340 mm, T2WI: TR 4500 ms, TE 80.0 ms, slice thickness 5.0 mm, slice spacing 0.5 mm, and FOV 200 mm ×240 mm. DCE scanning was conducted as follows: all patients underwent a plain scan before intravenous injection of the contrast agent Gd-DTPA at a dose of 0.1 mmol/kg and an injection speed of 2.0 mL/s, followed by fast gradient echo sequence dynamic scanning with the following parameters: TR 8 ms, TE 3.2 ms, slice thickness 3.0 mm, slice spacing 0.3 mm, and FOV 296 mm ×384 mm.

Next, an image post-processing workstation was employed to analyse the obtained images for making the diagnosis by two experienced physicians. Specifically, the area of interest of significantly enhanced lesions was selected, which was slightly smaller than lesions, and the areas with necrosis, hemorrhage, and calcification were avoided. Then a time signal-intensity curve (TIC) was plotted, and related parameters such as SI_max_, PH, the maximum linear slope and the ratio of maximum linear slope Slope^R^ were obtained. MRI signs^10^ were suspicious punctate enhancement signs in glands, pathological changes with irregular shapes, unclear boundaries, irregular annular enhancement signs and star-shaped edges, and glands with duct-like enhancement therein, branch-like parenchyma or pebble-like enhancement signs and a “fast in and out” curve shown on enhanced scanning images.

### Criteria for gland typing

With reference to the American College of Radiology, the mammary gland parenchyma was classified into 4 types based on the proportions of mammary gland parenchyma (fibro glandular tissues) and fat^11^: 1) adipose glands (mammary gland tissues are almost completely replaced by adipose tissues), 2) scattered fibrous glands (some thin fibrous glandular tissues are scattered continuously in mammary glands), 3) inhomogeneous and dense glands (mammary gland tissues are unevenly distributed, with uneven density), and 4) extremely dense glands (mammary gland tissues are evenly distributed, with uniform density).

### Statistical analysis

SPSS 22.0 software was employed for statistical analysis. Count data were represented as frequencies or rates, and the χ^2^ test was used for comparison between groups. Two-tailed P<0.05 indicated that the difference was statistically significant. With the results of the pathological diagnosis by biopsy as the “golden standard”, calculation was conducted for the sensitivity, specificity, accuracy, positive predictive value and negative predictive value of DCE-MRI and mammography for diagnosing breast cancer according to the following formulas: sensitivity = true positive/(true positive + false negative) ×100%, specificity = true negative/(false positive + true negative) ×100%, accuracy = (true positive + true negative)/(true positive + false positive + true negative + false negative) ×100%, positive predictive value = true positive/(true positive + false positive) ×100%, and negative predictive value = true negative/(true negative + false negative) ×100%. Multivariate logistic regression analysis was used for the sensitivity in diagnosing breast cancer. The significant level was set as α=0.05.

## Results

### Pathological diagnosis results

According to pathological diagnosis, malignant pathological changes were found in 47 of 93 patients with breast cancer, with a tumor lesion diameter of 0.5-1.8 (1.0±0.38) cm. In terms of the pathological type, there were 32 cases of ductal carcinoma in situ and 15 cases of invasive ductal carcinoma. As to the tumor-node-metastasis (TNM) stage, 30 cases were in TNM stage I, 10 cases were in stage II, and 7 cases were in stage III. Benign pathological changes were detected in the remaining 46 patients. The lesions were 0.3-1.7 (1.1±0.6) cm in diameter, and fibroma (n=21), lobular hyperplasia (n=13) and mastitis (n=12) were involved.

### Mammographic manifestations of breast cancer

The direct mammographic signs of malignant pathological changes were cluster calcification (n=23, 48.94%), small nodule shadows (n=10, 21.28%), star sign (n=7, 14.89%) and increased density shadows (n=7, 14.89%). In benign pathological changes, round high-density nodules with smooth edges indicated fibromas, villous high-density shadows and loss of normal structure of the breast suggested lobular hyperplasia, and irregular dense mass shadows with flamboyancy surrounded and blurred edges signified mastitis.

### DCE-MRI results

**Figure 1:**
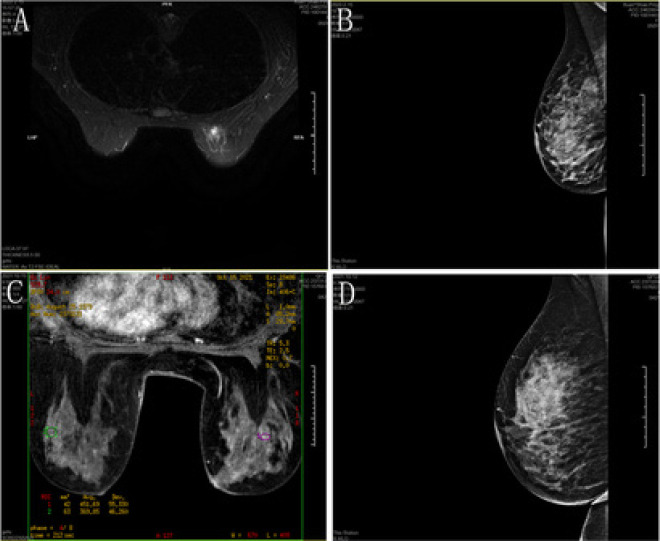
Typical case figures. A: DCE-MRI for breast cancer: Multiple dot-, sheet- and cord-like T2FS hyperechoic shadows can be observed, and nodules occupied the upper inner quadrant of the right breast, which had a size of about 18×14 mm, strong T2WI signal and weak T1WI signal. Post-enhanced scan showed obvious enhancement in the early stage. B: Mammography for breast cancer. C: DCE-MRI for benign breast lesion: Mammary glands on both sides were ACR3-type. There was strong patch- and cord-like T2fs signals in the areola and upper outer quadrant, and the signal with DWIb value of 500-800 was weakened. There was an irregular mass in the outer upper part of the right breast close to the axillary area, and the diffusion signal was enhanced with increasing b value. The enhanced scan showed rapid inflow and outflow enhancement, and the patch-like nodules in the mammary glands on both sides all exhibited inflow enhancement, without enlarged lymph nodes in both armpits. D: Mammography for benign breast lesion

Malignant pathological changes had an early enhancement rate of ≥60%, whereas benign pathological changes had an early enhancement rate of <60%. As to TIC type, malignant pathological changes were mainly type III, while benign pathological changes were mainly type I (Table 1). No statistically significant difference was found in SI-max and PH between benign and malignant pathological changes, while Slope and Slope^R^ were significantly larger in malignant pathological changes than those in benign malignant pathological changes (P<0.05) (Table 2). The typical case figures of mammography and DCE-MRI are exhibited in Figure 1.

**Table 1:** DCE-MRI results

Group	n	Early enhancement rate		TIC type		

	≥60%		<60%	I	II	III
Malignant pathological changes	47	38 (80.85)	9 (19.15)	5 (10.64)	7 (14.89)	35 (74.47)
Benign pathological changes	46	13 (28.26)	33 (71.74)	31 (67.39)	11 (23.91)	4 (8.70)
χ^2^		16.848			30.581	
P		<0.001			<0.001	

**Table 2:** Magnetic resonance parameters of benign and malignant pathological changes in breast

Group	n	SImax	PH	Slope	Slope^R^
Malignant pathological changes	47	298.17±58.82	143.95±23.47	62.54±25.12	11.65±4.50
Benign pathological changes	46	291.76±59.83	141.09±20.88	27.37±15.66	2.57±1.42
*t*		0.290	1.095	22.198	10.145
P		0.773	0.276	<0.001	<0.001

### Results of mammography, DCE-MRI and combined diagnosis as well as pathological diagnosis results in early-stage breast cancer detection

A total of 40 and 41 patients were diagnosed with malignant pathological changes by mammography and DCE-MRI, respectively. According to the combined diagnosis, 45 patients had malignant pathological changes. Compared to single diagnosis method, the combined diagnosis had significantly raised sensitivity, specificity, accuracy, positive predictive value and negative predictive value, and decreased rates of missed diagnosis and misdiagnosis (P<0.05) (Table 3 and 4).

**Table 3:** Results of mammography, DCE-MRI and combined diagnosis as well as pathological diagnosis results in early-stage breast cancer detection

Diagnostic method and result	Pathological diagnosis		Total
	Malignant	Benign	
Mammography			
Malignant	40	11	51
Benign	7	35	42
Total	47	46	93
DCE-MRI			
Malignant	41	10	51
Benign	6	36	42
Total	47	46	93
Combined diagnosis			
Malignant	45	2	47
Benign	6	40	46
Total	51	42	93

**Table 4:** Diagnostic efficiencies of mammography, DCE-MRI and combined diagnosis

Diagnostic method	Sensitivity	Specificity	Accuracy	Misdiagnosis rate	Missed diagnosis rate	Positive predictive value	Negative predictive value
Mammography	85.11 (40/47)	76.09 (35/46)	80.65 (75/93)	23..91 (11/46)	14.89 (7/47)	78.43 (40/51)	83.33 (35/42)
DCE-MRI	87.23 (41/47)	78.26 (36/46)	82.80 (77/93)	21.74 (10/46)	12.77 (6/47)	80.39 (41/51)	85.71 (36/42)
Combined	95.74 (45/47)	86.96 (40/46)	91.40 (85/93)	13.04 (6/46)	4.26 (2/47)	88.24 (45/51)	95.24 (40/42)
diagnosis							
χ^2^	6.946	9.693	12.613	10.518	11.935	7.956	8.245
P	0.019	<0.001	<0.001	<0.001	<0.001	<0.001	<0.001

### Diagnostic sensitivities at different pathological parameters

The diagnostic sensitivities of mammography, DCE-MRI and combined diagnosis were significantly different in the presence of different lesion diameter, type of breast glands and lesion distribution (P<0.05) (Table 5).

**Table 5:** Diagnostic sensitivities of mammography, DCE-MRI and combined diagnosis at different pathological parameters

Pathological parameter		Number of malignant lesions	Diagnostic sensitivity		Statistical value		P

		Mammography		DCE-MRI	Combined diagnosis		
Lesion diameter		47	3.47±0.98	2.33±0.49	1.71±0.25	57.6 24	<0.001
Type of mammary glands	Adipose	5	5 (100.00)	5 (100.00)	5 (100.00)	11.2 39	<0.001
	Scattered fibrous	17	15 (88.24)	15 (88.24)	17 (100.00)		
	Inhomogeneous and dense	14	12 (85.71)	13 (92.86)	13 (92.86)		
	Extremely dense	11	8 (72.73)	8 (72.73)	10 (90.91)		
Lesion distribution	Upper inner quadrant	12	10 (83.33)	11 (91.67)	12 (100.00)	9.05 8	<0.001
	Upper outer quadrant	23	21 (91.30)	21 (91.30)	22 (95.65)		
	Lower inner quadrant	10	8 (80.00)	8 (80.00)	9 (90.00)		
	Lower outer quadrant	2	1 (50.00)	1 (50.00)	2 (100.00)		

### Results of factors affecting sensitivity for diagnosing breast cancer

Univariate and multivariate logistic regression analyses were carried out for clinical data such as lesion diameter, type of breast glands, menstrual status, and lesion distribution, with the diagnosis result of breast cancer as the dependent variable (consistent with the pathological diagnosis result=0, inconsistent=1). The results revealed that lesion diameter was an independent risk factor affecting the sensitivity in breast cancer diagnosis (P<0.05) (Table 6).

**Table 6:** Results of factors affecting sensitivity for diagnosing breast cancer

Factor	Univariate			Multivariate		

	OR	95% CI	P	OR	95% CI	P
Lesion diameter	3.155	(1.462,3.247)	<0.001	2.578	(1.012,3.864)	0.009
Type of mammary glands	2.716	(1.450,3.476)	0.010	1.360	(1.085,1.879)	0.236
Menstrual status	2.972	(2.374,4.023)	0.023	1.834	(1.320,2.548)	0.549
Lesion distribution	2.469	(1.232,2.850)	0.018	1.332	(1.984,2.741)	0.813

## Discussion

Breast cancer is characterized by no obvious clinical symptoms in the early stage, inconspicuousness and high diffusion, which ranks first among all female malignancies in terms of incidence rate. Hence, early screening and diagnosis and timely intervention are of great significance to improving the survival rate and prognosis of patients with breast cancer.

As the first choice for screening breast cancer in clinical practice, mammography can decrease the mortality rate. Mammography is highly sensitive to microcalcification and exhibits high sensitivity and specificity in diagnosing early-stage breast cancer that presents only local incrassation and no evident clinical symptoms^12^. The direct signs of breast cancer in mammography are masses that are mostly solitary with high density, accompanied by different degrees of lobulation and burrs, which should be distinguished from benign lesions^13^. As a common sign in mammography, calcification is manifested as star sign, asymmetric density increases or local structural disorder, and there is no typical calcification or mass shadow of breast cancer^14^. However, due to a low-density resolution, mammography has poor sensitivity in the diagnosis of dense and small glands, fails to distinguish limited lobular hyperplasia and small adenomas with unclear margins, and has limitations in differentiating malignant from benign lesions^15^. Among the 47 patients with malignant pathological changes in this study, 40 were detected by mammography. The direct signs of malignant pathological changes were cluster calcification, small nodule shadows, star sign and increased density shadows. In benign pathological changes, round high-density nodules with smooth edges indicated fibromas, villous high-density shadows and loss of normal structure of the breast suggested lobular hyperplasia, and irregular dense mass shadows with flamboyancy surrounded and blurred edges signified mastitis. The diagnostic results of different mammary gland types showed that the detection rate of mammography for adipose malignant pathological changes was 100% and decreased to 72.73% for extremely dense malignant pathological changes. This is in agreement with the conclusion that the diagnostic sensitivity of mammography educes with increasing gland density^16^.

DCE-MRI, as a vital supplementary approach for breast imaging examination, has the advantages of multi-sequence, multi-parameter and multi-directional imaging, which is able to clearly display the morphological and hemodynamic characteristics of pathological changes in mammary glands^17^. In this study, among the 47 patients with malignant pathological changes, 41 were detected by DCE-MRI. The signs of breast cancer in DCE-MRI were irregular lesions with surrounding spiculation, and significantly enhanced satellite lesions were observed. Besides, the hemodynamic characteristics of breast cancer can also be employed as one of the references for diagnosis. An early enhancement rate lower than 60% suggests benign pathological changes, while an enhancement rate higher than 80% indicates malignant pathological changes. In the case of an enhancement rate of 60-80%, it is hard to determine the nature of pathological changes in tumors^18^. The early enhancement of TIC curve marks the speed of contrast agent in blood vessels flowing out to the extravascular intercellular space before the equilibrium period. More new vessels in tumors suggest lower permeability of the vessel wall and more obvious enhancement. The middle and late enhancement of the curve mainly reflects the internal and external equilibrium and outflow of blood in lesions. In most cases, Type I and III TIC curves indicate benign and malignant pathological changes, and a type II TIC curve suggests the presence of both benign and malignant pathological changes^19^. In this study, malignant pathological changes had an early enhancement rate of ≥60%, whereas benign pathological changes displayed an early enhancement rate of <60%. As to TIC type, malignant pathological changes were mainly type III, while benign pathological changes were mainly type I, being in line with previous findings. Moreover, Slope and SlopeR showed significant differences between benign and malignant lesions. Slope overlap was observed in benign and malignant tissues, whereas SlopeR usually displayed a normal distribution without overlap, with high value in diagnosing the nature of lesions^20^.

Leithner et al.^21^ reported that the sensitivity, specificity and coincidence rate of DCE-MRI for diagnosing benign and malignant pathological changes in breast cancer were 87.5%, 87.5%, and 86.9%, respectively. Additionally, Zhao et al.^22^ revealed that the sensitivity, specificity and accuracy of mammography combined with DCE-MRI in breast cancer diagnosis were 88.89%, 91.21% and 90.00%, respectively, significantly exceeding the diagnostic efficiency of a single examination method. In this study, the sensitivity, specificity and accuracy of combined diagnosis were 95.74%, 86.96%, and 91.40%, respectively, being significantly higher than those of a single diagnosis method. In addition, the missed diagnosis and misdiagnosis rates of combined diagnosis were significantly lower than those of a single diagnosis method. These results are basically in line with previous findings. On this basis, factors affecting the diagnostic sensitivity in breast cancer were investigated based on the clinicopathological characteristics of patients. The average diameters of breast lesions detected by different diagnostic methods were different. The lesion diameter obtained in mammography combined with DCE-MRI was 1.71 cm, and the maximum tumor diameter was an independent risk factor for the diagnostic sensitivity.

In conclusion, both mammography and DCE-MRI play essential roles in the early diagnosis of breast cancer, and their combination can augment the diagnostic efficiency, reduce the rates of missed diagnosis and misdiagnosis, and provide an objective basis for clinical treatment. However, in this study, only the nature (namely malignant or benign) of pathological changes in breast cancer was diagnosed, while the specific pathological type was not identified. Hence, the value of mammography combined with DCE-MRI in diagnosing different pathological types of breast cancer should be further explored.
